# Microcirculatory perfusion disturbances following cardiopulmonary bypass: a systematic review

**DOI:** 10.1186/s13054-020-02948-w

**Published:** 2020-05-13

**Authors:** Matthijs M. den Os, Charissa E. van den Brom, Anoek L. I. van Leeuwen, Nicole A. M. Dekker

**Affiliations:** 1 Department of Anesthesiology, Amsterdam UMC, Vrije Universiteit Amsterdam, Amsterdam Cardiovascular Sciences, De Boelelaan 1117, 1081 HV Amsterdam, The Netherlands; 2 Department of Physiology, Amsterdam UMC, Vrije Universiteit Amsterdam, Amsterdam Cardiovascular Sciences, Amsterdam, The Netherlands; 3 Department of Cardiothoracic surgery, Amsterdam UMC, Vrije Universiteit Amsterdam, Amsterdam Cardiovascular Sciences, Amsterdam, The Netherlands

**Keywords:** Microcirculation, Microcirculatory perfusion, Cardiopulmonary bypass, Cardiac surgery, Sublingual, Capillary perfusion

## Abstract

**Background:**

Microcirculatory perfusion disturbances are associated with increased morbidity and mortality in patients undergoing cardiac surgery with cardiopulmonary bypass (CPB). Technological advancements made it possible to monitor sublingual microcirculatory perfusion over time. The goal of this review is to provide an overview of the course of alterations in sublingual microcirculatory perfusion following CPB. The secondary goal is to identify which parameter of sublingual microcirculatory perfusion is most profoundly affected by CPB.

**Methods:**

PubMed and Embase databases were systematically searched according to PRISMA guidelines and as registered in PROSPERO. Studies that reported sublingual microcirculatory perfusion measurements before and after onset of CPB in adult patients undergoing cardiac surgery were included. The primary outcome was sublingual microcirculatory perfusion, represented by functional capillary density (FCD), perfused vessel density (PVD), total vessel density (TVD), proportion of perfused vessels (PPV), and microvascular flow index (MFI).

**Results:**

The search identified 277 studies, of which 19 fulfilled all eligibility criteria. Initiation of CPB had a profound effect on FCD, PVD, or PPV. Seventeen studies (89%) reported one or more of these parameters, and in 11 of those studies (65%), there was a significant decrease in these parameters during cardiac surgery; the other 6 studies (35%) reported no effect. In 29% of the studies, FCD, PVD, or PPV normalized by the end of cardiac surgery, and in 24% percent of the studies, this effect lasted at least 24 h. There was no clear effect of CPB on TVD and a mixed effect on MFI.

**Conclusion:**

CPB during cardiac surgery impaired sublingual microcirculatory perfusion as reflected by reduced FCD, PVD, and PPV. Four studies reported this effect at least 24 h after surgery. Further research is warranted to conclude on the duration of CPB-induced microcirculatory perfusion disturbances and the relationship with clinical outcome.

**Trial registration:**

PROSPERO, CRD42019127798

## Background

Microcirculatory perfusion disturbances are commonly reported in patients undergoing cardiac surgery with cardiopulmonary bypass (CPB). Cardiac surgery with CPB is associated with risk of morbidity such as mediastinitis, permanent stroke, acute kidney injury, and acute lung injury [1, 2]. Microcirculatory perfusion disturbances are additionally associated with increased morbidity and mortality in the ICU in patients with cardiogenic shock or sepsis [3, 4]. Interestingly, however, there appears to be an uncoupling of macrocirculatory and microcirculatory hemodynamics [5], meaning that sustainment of systemic hemodynamic parameters during surgery does not guarantee adequate microcirculatory perfusion. Therefore, real-time imaging might be a valuable tool to monitor alterations in microcirculatory perfusion in patients undergoing cardiac surgery with CPB and guide interventions in the perioperative period.

Technological advancements made it possible to monitor sublingual microcirculatory perfusion. Since 1999, the invention of the orthogonal polarization spectral (OPS) imaging technique allowed researchers to study the microcirculatory perfusion in real time [6]. Technological upgrades have resulted in a second-generation side-stream dark field (SDF) imaging device and a third-generation incidence dark field (IDF) imaging device. With each generation, image quality has improved dramatically, making it possible to visualize more microvessels [7]. Naturally with improved image quality, the methods to analyze images have evolved as well. For the first generation of devices, semi-quantitative analyzation methods were introduced, whereas now it is possible to analyze each individual vessel in an image over time. To structure research, standards for image quality and reporting have been developed by a panel of experts and there is debate on which parameters are superior to monitor microcirculatory perfusion [8].

Over the past decade, a lot of research has been conducted in this promising field. However, to our knowledge, no comprehensive review has been published specifically about the effects of CPB on microcirculatory perfusion. The goal of this review is to provide an overview of the course of alterations in sublingual microcirculatory perfusion in cardiac surgery patients following CPB. The second aim is to identify which microcirculatory perfusion parameters are most profoundly affected by CPB in patients undergoing cardiac surgery.

## Methods

### Protocol and registration

Details of the protocol for this review were preregistered at the International prospective register of systematic reviews, PROSPERO with registration number CRD42019127798. Systematic review methodology is reported according to the Preferred Reporting Items for Systematic Reviews and Meta-Analyses (PRISMA) guidelines [9].

### Eligibility criteria

This systematic review included clinical studies that measured sublingual microcirculatory perfusion in patients undergoing elective cardiac surgery with CPB. Any duration and protocol of CPB were included ((mild) hypothermia, pulsatile flow, non-pulsatile flow). Study protocols with any type of elective cardiac surgery were eligible for inclusion. A baseline measurement of sublingual microcirculatory perfusion before onset of CPB was required as control. Exclusion criteria were in vitro studies, animal studies, pediatric subjects, and emergency surgery.

### Search strategy

On March 7, 2019, PubMed and Embase were searched for any publication that reported sublingual microcirculatory perfusion in adult patients undergoing cardiac surgery with CPB. The search strategy was based on a combination of the following search terms: “cardiopulmonary bypass,” “cardiac surgery,” and “microcirculatory perfusion” (Additional file 1). Reference lists of all the full texts were screened for further eligible studies. Any study that reported sublingual microcirculatory perfusion in combination with CPB in adult patients undergoing cardiac surgery was of interest. All study designs were eligible for inclusion, but case reports, conference abstracts, letters, editorials, and reviews were excluded.

### Study selection

Two independent reviewers (MO and ND) scanned all titles and abstracts to identify studies that potentially met inclusion criteria. Full texts were obtained for studies that appeared to be of interest. Eligible studies were identified by two reviewers from reading full texts (MO and ND). Disagreements between the reviewers were resolved by discussion with involvement of a third reviewer (CvdB).

### Data extraction

Data extraction was performed by one reviewer (MO) and confirmed by another (ND). Data were extracted with regard to the study characteristics and design, demographic data of patients, anesthetic protocol, CPB protocol, type of surgery, and surgery characteristics such as duration, time on CPB, and cross-clamp time. Details regarding monitoring device of microcirculatory perfusion (OPS, SDF, or IDF), microcirculatory perfusion parameters, and clinical outcomes were also extracted (see Additional file 2).

### Quality assessment

Risk of bias was assessed independently by two reviewers (MO and ND) with the NIH quality assessment tool [10]. The applicable NIH quality assessment tool was used depending on study designs (RCT or observational study). The NIH quality assessment tool assesses selection, performance, detection, attrition, and reporting biases through a set list of yes or no questions. The aggregated scores are not meant as summary judgment of quality but help reviewers to assess the aforementioned forms of bias.

### Definition of microcirculatory perfusion parameters

The primary outcome of this review was sublingual microcirculatory perfusion measured by orthogonal polarization spectral imaging (OPS) or its successors, side-stream dark field imaging (SDF) or incidence dark field imaging (IDF). These devices are based on the principle that scattered green light is absorbed by hemoglobin of red blood cells, thereby visualizing flowing red blood cells, subsequently indirectly visualizing of arterioles and venules. A minimum of three recordings per time point were required to account for the intrinsic variability of the microcirculation and to correct for exclusion of recordings due to low image quality [11].

The following parameters can be obtained from the videos: functional capillary density (FCD), perfused vessel density (PVD), total vessel density (TVD), small vessel density (SVD), vessel density (VD), proportion of perfused vessels (PPV), and microvascular flow index (MFI).

FCD is defined as the total length of microvessels relative to the image size that exhibit normal flow during the length of the recording. Normal flow is defined as continuous flow through a microvessel during the length of a recording, absent flow as no flow during the recording, and intermittent flow when at least 50% of the time no flow is observed. PVD is an estimate of the FCD and can be calculated by multiplying the total number of vessels with the proportion of perfused vessels.

TVD is defined as the total length of the vessels visible in an image relative to the size of the image (mm/mm^2^). SVD is defined as the total length of all vessels with a diameter ≤ 25 μm visible in an image relative to the size of the image (mm/mm^2^). VD is defined as the number of vessels crossing an arbitrary grid of three by three lines, drawn on the video, relative to the length of the lines (n/mm). PPV is calculated as follows: 100 × (total number of vessels − [no flow + intermittent flow]/total number of vessels).

MFI is a score based on determination of the predominant type of flow in four quadrants of a recording. Flow is characterized as absent (0), intermittent (1), sluggish (2), or normal (3). The values of the four quadrants are averaged.

### Data analysis

Due to heterogeneity of studies, a narrative synthesis of the effect of CPB on microcirculatory perfusion was performed. As different microcirculatory perfusion monitoring devices employing different techniques (OPS, SDF) were used, absolute values of microcirculatory perfusion could not be compared. Therefore, only significant increases or decreases in microcirculatory perfusion parameters following CPB compared with pre-CPB measurements per included study were reported.

## Results

**Fig. 1:**
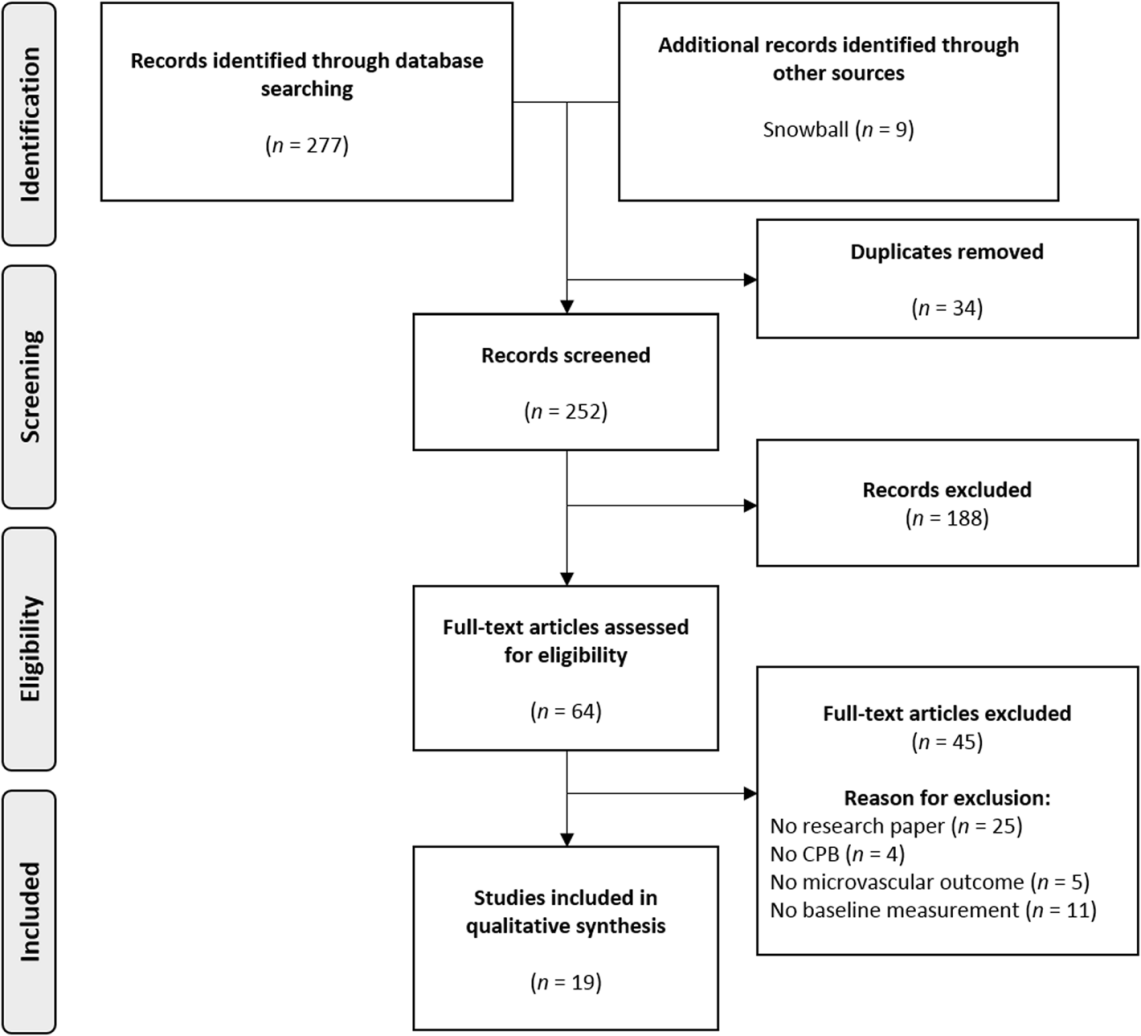
PRISMA flow diagram representing the flowchart of study selection. PRISMA, Preferred Reporting Items for Systematic Reviews and Meta-Analyses

The study selection is presented in a PRISMA diagram (Fig. 1). The initial search identified 277 records, of which 252 were screened and 188 studies were excluded. Finally, 64 full texts were examined and 45 were excluded based on conference abstracts (*n* = 20), absence of CPB (*n* = 4), absence of microcirculatory perfusion measurement (*n* = 5), missing pre-operative measurement (*n* = 11), and reviews (*n* = 5). Finally, 19 studies were included that recorded perioperative measurements of sublingual microcirculatory perfusion parameters in adult patients undergoing cardiac surgery with CPB [12–30].

### Study characteristics

The 19 studies included in this review consisted of 9 randomized controlled trials and 10 observational studies. All studies were published between 2007 and 2019 by 14 individual authors. Research was performed in 9 countries: Belgium (*n* = 1), Canada (*n* = 3), Denmark (*n* = 1), Egypt (*n* = 1), Germany (*n* = 3), Netherlands (*n* = 8), Turkey (*n* = 1), and Uruguay (*n* = 1).

### Patient characteristics

Patient characteristics are listed in Additional file 3. A total of 584 patients participated in these studies. A variety of interventions were studied such as mode of CPB, type of anesthesia, or differences between types of surgery. At least 398 (68%) participants were male (one study did not report gender). Most common comorbidities were hypertension (57%) and diabetes mellitus, type not specified (26%). None of the included studies reported significant differences between patient characteristics at baseline.

### Risk of bias

The full risk of bias assessment is summarized in Additional file 4. Only 37% of all 19 included studies reported sample size calculation. Of the nine randomized controlled trials, 22% reported no clear method of randomization. In 89% of the randomized controlled trials, caregivers were not blinded from the intervention (type of perfusion, anesthetic medication). In the ten observational studies, 40% of the studies did not report whether outcome assessors were blinded to the interventions. Only one study raised concerns regarding outcome reporting [24].

### Functional capillary density, perfused vessel density, and proportion of perfused vessels

Alterations in FCD, PVD, and PPV are presented in Table 1. A distinction is made between the direct effect of CPB on microcirculatory perfusion parameters during cardiac surgery and the effect of CPB on microcirculatory perfusion parameters postoperatively at the ICU or the ward. Four out of 19 studies (21%) [12, 13, 16, 17] reported the FCD and all four studies showed a significant decrease of FCD during CPB compared to pre-CPB measurements. Eight studies (42%) reported PPV, of which 63% [14, 15, 18–20] showed a reduction in PPV following CPB compared to baseline pre-CPB. The other studies that reported PPV (37%) [21–23] reported no significant effect of CPB on PPV. Eleven studies (57%) reported PVD, of which 64% [14, 15, 20, 24–27] showed a significant decrease in PVD and 36% [21–23, 28] showed no effect of CPB on PVD. In total, 17 out of 19 studies reported FCD, PPV or PVD and 77% of these studies showed a decrease in one or more of these microcirculatory perfusion parameters during cardiac surgery with CPB.

**Table 1:** Perioperative changes in sublingual perfused vessel density (PVD), functional capillary density (FCD), and proportion of perfused vessels (PPV) per study

Study	***n***	Technique	Study groups	Pre-CPB	CPB		Post-CPB				
				Induction	Onset of CPB	End of CPB	Post-CPB	ICU	24 h after surgery	48 h after surgery	72 h after surgery
Atasever et al. [12]	24	SDF	CABG	–	↓ FCD	–	–	–	–	–	–
Bauer et al. [13]	47	OPS	CS	–	~ FCD	↓ FCD	~ FCD	–	–	–	–
Bienz et al. [28]	16	SDF	CABG	~ PVD	~ PVD	~ PVD	~ PVD	~ PVD	–	–	–
De Backer et al. [14]	9	OPS	CS	↓ PVD/PPV	↓ PVD/PPV	–	↓ PVD/PPV	↓ PVD/PPV	~ PVD, ↓ PPV	–	–
Dekker et al. [15]	17	SDF	CABG	~ PVD/PPV	↓ PVD/PPV	–	↓ PVD/PPV	↓ PVD/PPV	↓ PVD/PPV	–	↓ PVD/PPV
Donndorf et al. [16]	40	OPS	CABG	–	↓ FCD	↓ FCD	~ FCD	–	–	–	–
			CABG with MECC	–	↓ FCD	~ FCD	~ FCD	–	–	–	–
Donndorf et al. [17]	20	OPS	AVR	–	↓ FCD	↓ FCD	~ FCD	–	–	–	–
			AVR with MECC	–	↓ FCD	↓ FCD	~ FCD	–	–	–	–
Holmgaard et al. [21]	30	SDF	CABG with HMAP	–	~ PVD/PPV	–	~ PVD/PPV	–	–	–	–
			CABG with LMAP	–	~ PVD/PPV	–	~ PVD/PPV	–	–	–	–
Koning et al. [24]	33	SDF	CABG non-pulsatile CPB	–	~ PVD	~ PVD	↓ PVD	↓ PVD	–	–	–
			CABG pulsatile CPB	–	~ PVD	~ PVD	~ PVD	~ PVD	–	–	–
Koning et al. [31]	13	SDF	CABG		~ PVD	↓ PVD	↓ PVD	↓ PVD	–	–	–
Koning et al. [26]	24	SDF	CABG non-pulsatile CPB	–	↓ PVD	–	↓ PVD	–	–	–	–
			CABG pulsatile CPB	–	↓ PVD	–	~ PVD	–	–	–	–
Mohamed et al. [22]	70	SDF	CABG regular anesthesia	–	~ PVD/PPV	–	~ PVD/PPV	–	–	–	–
			CABG dexmedetomidine	–	~ PVD/PPV	–	~ PVD/PPV	–	–	–	–
O’Neil et al. [18]	20	OPS	CS with non-pulsatile CPB	–	~ PPV	~ PPV	↓ PPV	↓ PPV	↓ PPV	↓ PPV	–
			CS with pulsatile CPB	–	~ PPV	~ PPV	~ PPV	~ PPV	~ PPV	~ PPV	–
O’Neil et al. [19]	20	OPS	CS with non-pulsatile CPB	–	~ PPV	↓ PPV	↓ PPV	–	↓ PPV	–	–
			CS with pulsatile CPB	–	~ PPV	~ PPV	~ PPV	–	~ PPV	–	–
Özarslan et al. [20]	30	OPS	CABG sevoflurane	~ PVD/PPV	~ PVD, ↓ PPV	–	~ PVD/PPV	–	~ PVD/PPV	–	–
			CABG isoflurane	~ PVD/PPV	↓ PVD/PPV	–	~ PVD/PPV	–	~ PVD/PPV	–	–
			CABG desflurane	~ PVD/PPV	↓ PVD/PPV	–	~ PVD/PPV	–	~ PVD/PPV	–	
Prestes et al. [23]	22	SDF	CS	–	~ PVD/PPV	~ PVD/PPV	~ PVD/PPV	–	–	–	–
Yuruk et al. [27]	20	SDF	CABG	–	↓ PVD	–	~ PVD	–	–	–	–
			CABG with MECC	–	~ PVD	–	~ PVD	–	–	–	–

A downward facing arrow (↓) represents a significant decrease compared to baseline measurement, ~ represents no significant change compared to baseline measurement. *FCD* functional capillary density, *PVD* perfused vessel density, *PPV* proportion of perfused vessels, *CPB* cardiopulmonary bypass, *SDF* side-stream dark field imaging, *OPS* orthogonal polarization spectral imaging, *n* number of participants, *h* hours, *CS* cardiac surgery, *CABG* coronary artery bypass grafting, *MECC* minimal extracorporeal circulation, *LMAP* low mean arterial pressure; HMAP, high mean arterial pressure

Interestingly, the timing and duration of the decrease in FCD, PPV, or PVD varied across studies, depending on the follow-up period. Five studies found that the CPB-induced decrease of FCD, PPV, or PVD had resolved by the end of surgery [13, 16, 17, 20, 27]. In four studies, the duration of the CPB-induced effect on microcirculatory perfusion remained unclear, as the FCD, PPV, or PVD had not returned to baseline at the last reported measurement [12, 24–26]. Eight studies followed patients postoperatively of which three studies had a follow-up of 24 h [14, 19, 20], one study of 48 h [18], and one study of 72 h [15]. Four of these studies reported a CPB-induced reduction in FCD, PPV, or PVD for at least 24 h after surgery [14, 15, 18, 19].

### Total vessel density, vessel density, and small vessel density

The effect of CPB on TVD, VD, or SVD is presented in Table 2. Of the 19 included studies, 42% (*n* = 8) reported one or more of these parameters. Four studies reported no effect at all of initiation of CPB on TVD, VD, or SVD. One study reported a lasting decrease of TVD after initiation of CPB, compared to pre-CPB measurements that had not returned to baseline after arrival on the ICU [25]. One study found a temporary decrease in TVD after the start of CPB, which had normalized by the end of surgery [20]. One study observed a temporary increase in SVD after start of CPB, which returned to baseline before the end of surgery. Also, in this study, no effect of CPB on TVD was reported [28].

**Table 2:** Perioperative changes in sublingual total vessel density (TVD), small vessel density (SVD), and vessel density (VD) per study

Study	***n***	Technique	Study groups	Pre-CBP	CPB		Post-CPB		
				Induction	Onset of CPB	Late phase of CPB	Post-CPB	ICU	24 h after surgery
Bienz et al. [28]	16	SDF	CABG	~ TVD	~ TVD	~ TVD	~ TVD	~ TVD	–
			CABG	~ SVD	~ SVD	↑ SVD	~ SVD	~ SVD	–
De Backer et al. [14]	9	OPS	Cardiac surgery with CPB	~ VD	~ VD	–	~ VD	~ VD	~ VD
Holmgaard et al. [21]	30	SDF	CABG with HMAP	–	~ TVD	–	~ TVD	–	–
			CABG with LMAP	–	~ TVD	–	~ TVD	–	–
Koning et al. [24]	33	SDF	CABG non pulsatile CPB	–	~ TVD	~ TVD	~ TVD	~ TVD	–
			CABG pulsatile CPB	–	~ TVD	~ TVD	~ TVD	~ TVD	–
Koning et al. [31]	13	SDF	CABG	–	↓ TVD	↓ TVD	↓ TVD	↓ TVD	–
Mohamed et al. [22]	70	SDF	CABG with regular anesthesia	–	~ TVD	–	~ TVD	–	–
			CABG dexmed anesthesia	–	~ TVD	–	↓ TVD	–	–
Özarslan et al. [20]	30	OPS	CABG sevoflurane anesthesia	~ TVD	↓ TVD	–	~ TVD	–	~ TVD
			CABG isoflurane anesthesia	~ TVD	↓ TVD	–	~ TVD	–	~ TVD
			CABG desflurane anesthesia	~ TVD	~ TVD	–	~ TVD	–	~ TVD
Prestes et al. [23]	22	SDF	CS with CPB	–	~ VD	~	~ TVD	–	–

A downward facing arrow (↓) represents a significant decrease compared to baseline measurement. An upward facing arrow (↑) represents a significant increase compare to baseline measurement. ~ represents no significant change compared to baseline value. *TVD* total vessel density, *VD* vessel density, *SMD* small vessel density, *CPB* cardiopulmonary bypass, *SDF* side-stream dark field imaging, *OPS* orthogonal polarization spectral imaging, *n* number of participants, *h* hours, *CS* cardiac surgery, *CABG* coronary artery bypass grafting, *LMAP* low mean arterial pressure, *HMAP* high mean arterial pressure

### Microvascular flow index

Results for MFI are presented in Table 3. A total of 9 out of 19 studies (47%) reported the MFI. Three studies showed a significant decrease in MFI compared to baseline after initiation of CPB during cardiac surgery [22, 24, 29]. In contrast, two studies observed an increase in MFI compared to baseline after initiation of CPB during surgery [20, 23]. The four remaining studies observed no effect of CPB on MFI during cardiac surgery. However, two of these studies reported a significant decrease of MFI at the follow-up on the ICU compared to baseline [25, 30]. It is unclear how long the effect of CPB on MFI lasts. In four studies that reported a CPB-induced change in MFI, MFI had not returned to baseline at the last available measurement [22–25].

**Table 3:** Perioperative changes in sublingual microvascular flow index (MFI) per study

Study	***n***	Technique	Study groups	Pre-CPB	CPB		Post-CPB		
				Induction	Onset of CPB	Late phase of CPB	Post-CPB	ICU	24 h after surgery
den Uil et al. [29]	25	SDF	CS with CPB	~ MFI	~ MFI	–	~ MFI	~ MFI	–
			CS with CPB (vessels 25–50 μm)	~ MFI	↓ MFI	–	~ MFI	~ MFI	–
			CS with CPB (vessels 50–100 μm)	~ MFI	~ MFI	–	~ MFI	~ MFI	–
Holmgaard et al. [21]	30	SDF	CABG with HMAP	–	~ MFI	–	~ MFI	–	–
			CABG with LMAP	–	~ MFI	–	~ MFI	–	–
Koning et al. [24]	33	SDF	CABG non pulsatile CPB	–	~ MFI	↓ MFI	↓ MFI	↓ MFI	–
			CABG pulsatile CPB	–	~ MFI	~ MFI	~ MFI	~ MFI	–
Koning et al. [31]	13	SDF	CABG	–	~ MFI	~ MFI	~ MFI	↓ MFI	–
Koning et al. [30]	18	SDF	CABG	–	~ MFI	–		↓ MFI	–
Mohamed et al. [22]	70	SDF	CABG regular anesthesia	–	↓ MFI	–	↓ MFI	–	–
			CABG dexmed anesthesia	–	↓ MFI	–	~ MFI	–	–
Özarslan et al. [20]	30	OPS	CABG sevoflurane anesthesia	~ MFI	~ MFI	–	~ MFI	–	~ MFI
			CABG isoflurane anesthesia	~ MFI	↓ MFI	–	~ MFI	–	~ MFI
			CABG desflurane anesthesia	~ MFI	~ MFI	–	~ MFI	–	~ MFI
Prestes et al. [23]	22	SDF	Cardiac surgery with CPB	–	~ MFI	↓ MFI	↑ MFI	–	–
Yuruk et al. [27]	20	SDF	CABG	–	~ MFI	–	~ MFI	–	–
			CABG with MECC	–	~ MFI	–	~ MFI	–	–

A downward facing arrow (↓) represents a significant decrease compared to baseline measurement. An upward facing arrow (↑) represents a significant increase compare to baseline measurement. ~ represents no significant change compared to baseline value. *MFI* microvascular flow index, *CPB* cardiopulmonary bypass, *SDF* side-stream dark field imaging, *OPS* orthogonal polarization spectral imaging, *n* number of participants, *h* hours, *CS* cardiac surgery, *CABG* coronary artery bypass grafting, *MECC* minimal extracorporeal circulation, *LMAP* low mean arterial pressure group, *HMAP* high mean arterial pressure group

### Clinical outcome

Eight out of the 19 included studies provided any information on clinical outcomes of patients [16, 17, 19, 23, 24, 27, 29, 30]. One study reported two patient deaths; both showed impaired microcirculatory perfusion measured as MFI [29]. In two studies by the same group, one death was reported in each study; however, no information on microcirculatory perfusion measurements were provided [16, 17]. Two studies reported no major complications [24, 27]. In one study, 2 cases of acute kidney injury (AKI) were reported of which one patient died and both patients had a decreased MFI postoperatively when compared to preoperative values [30]. Another study found significantly higher creatinine values in the non-pulsatile perfusion group, which was correlated to significant microvascular alterations [19]. One group reported major complications in five patients without specifying the type of complication. They found that patients who developed postoperative complications showed increased hyperdynamic capillaries compared to patients without complications [23].

## Discussion

Cardiopulmonary bypass during cardiac surgery impairs sublingual microcirculatory perfusion. CPB-induced microcirculatory perfusion disturbances were most profoundly observed by changes in functional capillary density (FCD), perfused vessel density (PVD), or proportion of perfused vessels (PPV), but not total vessel density (TVD), small vessel density (SVD), vessel density (VD), or microvascular flow index (MFI). FCD, PVD, and PPV appear to remain disturbed throughout the entire surgical procedure; however, the exact duration of these microcirculatory perfusion alterations remains unclear. Further research is warranted to conclude on the duration of CPB-induced microcirculatory perfusion disturbances and the relationship with clinical outcome.

CPB-induced microcirculatory perfusion disturbances as represented by a decrease in FCD, PVD, or PPV were found in 77% of the studies. FCD and PVD are both a function of TVD and PPV. As TVD was not affected by CPB in most studies, these results suggests that the observed reductions in FCD and PVD were mainly the result of a reduced number of perfused vessels. Moreover, as a decrease in FCD, PVD, or PPV was mostly absent in patients undergoing off-pump CABG, the observed microcirculatory perfusion disturbances are likely a true CPB effect [12, 25, 26].

**Fig. 2:**
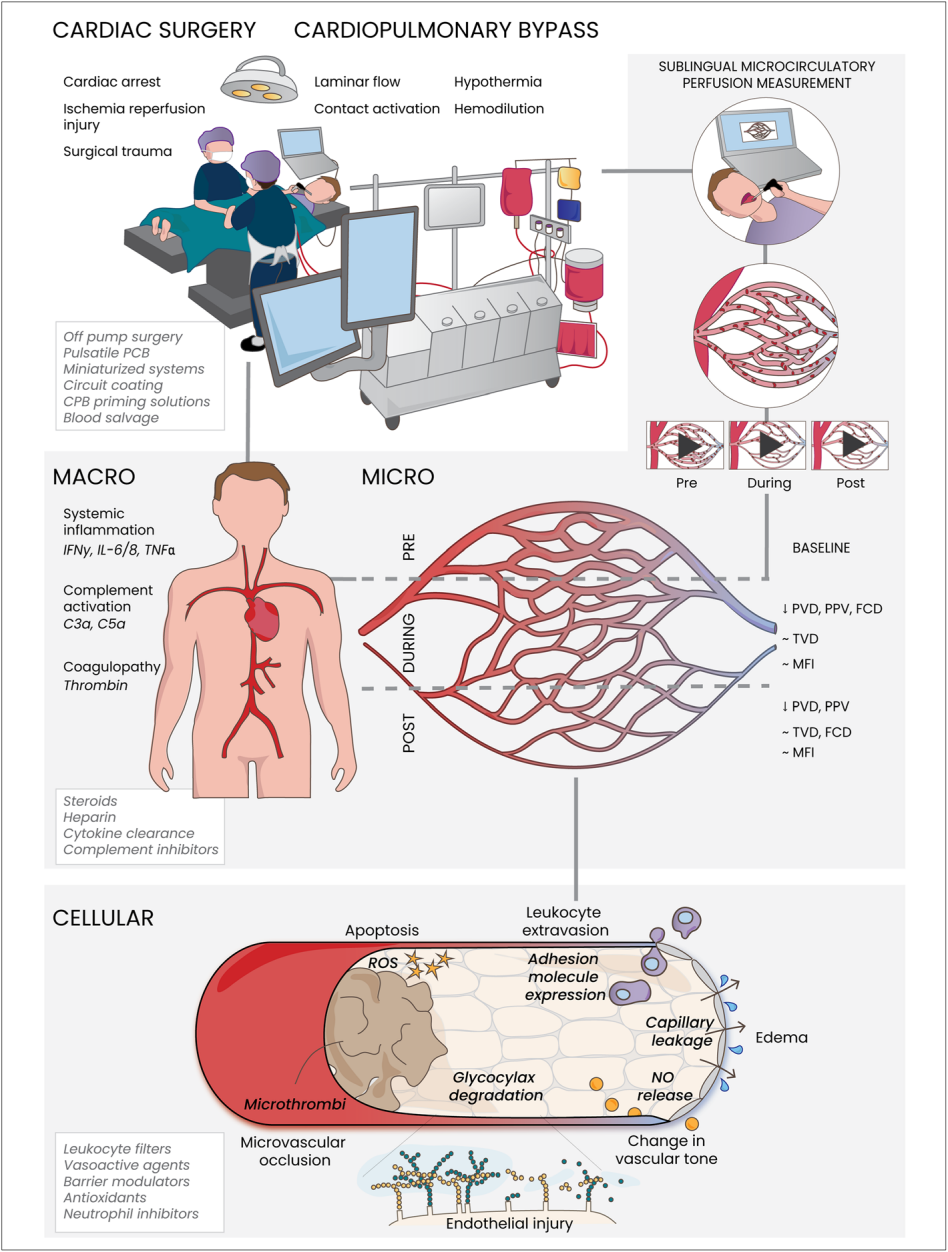
Summary figure. Cardiac surgery with cardiopulmonary bypass impairs microcirculatory perfusion, which is monitored sublingually in patients in the perioperative period. Onset of cardiopulmonary bypass reduces sublingual microcirculatory perfusion reflected by functional capillary density (FCD), proportion of perfused vessels (PPV), and perfused vessel density (PVD) compared to baseline, whereas total vessel density (TVD) remained unaltered. The effect of cardiopulmonary bypass on microvascular flow index (MFI) differed between studies. Pathophysiological mechanisms include systemic inflammation, and activation of complement and coagulation, which causes shedding of the endothelial protective glycocalyx layer leading to endothelial injury. In addition, release of barrier disruptive mediators induce endothelial barrier disruptive signaling, resulting in capillary leakage and edema formation. Activation of the endothelium stimulates the release of nitric oxide (NO), affecting vascular tone and systemic blood pressure. Moreover, induction of endothelial adhesion molecule expression increases leucocyte rolling and extravasation. Also, activation of polymorphonuclear neutrophils causes the release of reactive oxygen species (ROS), contributing to tissue injury. Activation of platelets and coagulation are associated with the formation of microthrombi and microvascular occlusion. Collectively, these mechanisms impair microcirculatory perfusion and contribute to organ injury following cardiac surgery with cardiopulmonary bypass, with clinical and experimental treatment strategies presented in italic in white boxes. IL, interleukin; IFNy, interferon gamma; TNFα, tumor necrosis factor alpha

In contrast, no effect of CPB on total vessel density (TVD), small vessel density (SVD), or vessel density (VD) was observed. These observations are in line with recent experimental and theoretical insights [30, 32, 33], suggesting that CPB does not necessarily affect the absolute number of microvessels, but mainly impairs microcirculatory red blood cell flow patterns, as reflected by a reduced number of perfused vessels (PVD). CPB-associated factors such as hemodilution, contact activation, and the induction of a systemic inflammatory response are thought to impair microcirculatory perfusion by affecting both transport and diffusion of oxygen at the microvascular level. Pathophysiological mechanisms include glycocalyx degradation and endothelial, platelet, and leucocyte activation leading to increased endothelial permeability and edema formation, leucocyte extravasation, and microthrombi formation (Fig. 2). These pathophysiological mechanisms involved in cardiopulmonary bypass-associated microvascular alterations and microcirculatory perfusion disturbances were previously discussed in detail [31, 34].

The effect of CPB on microvascular flow index (MFI) could not be clearly defined. This may be partly explained by the modified MFI criteria used in some studies, with an extra category for hyperdynamic flow confounding the results [20, 23]. The rationale is that at a slow, sluggish flow, oxygenation is not impaired due to the increased transit time for oxygen exchange, whereas a hyperdynamic flow with supranormal flow velocities of red blood cells may indicate impaired oxygen offloading [30]. However, sluggish flow might be detrimental through various other mechanisms (waste removal, impaired nutrient supply) although no literature yet exists on this subject to our knowledge. Importantly, the MFI, as currently constituted, does recognize one type of flow as abnormal (sluggish), yet does not account for another type of abnormal flow (hyperdynamic). Additionally, MFI predominantly assesses overall flow characteristics and lacks the precision to be used as a surrogate for microcirculatory perfusion. We therefore discourage the use of MFI in a research setting since it is inferior to PPV, TVD, and PVD to assess microcirculatory perfusion.

Yet, MFI remains a popular parameter in microcirculatory research, as it the easiest and fastest microcirculatory variable to determine, thereby allowing its use for point of care monitoring. Interestingly, abnormal baseline MFI of 2.6 or lower combined with tachycardia was found to be a good predictor of mortality in an everyday ICU setting [35]. This finding was replicated in the microDAIMON study, where the authors noted that daily offline analysis of PPV, TVD, and PVD was time consuming and had limited predictive value [36]. However, this study also showed that daily MFI changes did not relate to clinical outcomes limiting the use of MFI as a predictor at baseline only. In contrast, in two other studies with highly selected populations, day to day monitoring of microvascular parameters correlated with outcome [37, 38]. In conclusion, further research in sufficiently large study populations is necessary to determine the value of day to day microcirculatory monitoring. Until fully automated image analysis software becomes available, MFI may be of interest as the most easy microvascular perfusion parameter to monitor.

Factors such as anesthesia, type of surgery, and type of blood flow during CPB may influence the effect of CPB on sublingual microcirculatory perfusion. Multiple studies included in this review specifically measured the effect of certain types of anesthesia or reported pre and post induction of anesthesia measurements. Interestingly, only one study by De Backer et al. found that anesthesia contributed to a significant decrease in microcirculatory perfusion, but that this effect was transient [De Backer]. In contrast, none of the other studies that reported pre and post induction measurements reported similar findings [15, 20, 28, 29]. It was previously shown that propofol transiently reduced microcirculatory perfusion in 15 healthy young females that underwent oocyte retrieval under propofol sedation [39]. Possibly, these phenomena may explain the opposite findings by De Backer and the other studies; however, the exact propofol doses administered in the study by De Backer et al. were not reported.

The effect of volatile anesthetic agents on outcome in cardiac surgery remains a topic of debate [40]. Özarslan et al. compared the effect of various volatile anesthetics agents on sublingual microcirculatory perfusion and showed that sevoflurane compared to isoflurane and desflurane reduced microcirculatory perfusion during cardiac surgery, as reflected by a significantly lower PPV in small vessels during CPB. This effect was transient, as it had resolved by the end of surgery. Although anesthesia may affect microcirculatory perfusion, currently available data suggests this effect to be subtle and transient by nature.

Traditionally, CPB generates a non-pulsatile blood flow and this non-physiological blood flow may exhibit adverse effects on microcirculatory perfusion. In the present review, four studies compared a pulsatile blood flow during CPB with a non-pulsatile blood flow [19, 20, 24, 26]. Both studies of Koning et al. reported a restoration of microcirculatory perfusion following weaning from CPB with pulsatile flow compared to non-pulsatile flow, whereas both studies of O’Neill reported preservation of microcirculatory perfusion during CPB with pulsatile flow compared to non-pulsatile flow. Despite extensive literature, the question whether pulsatile flow during CPB may be superior to non-pulsatile flow remains unanswered [41, 42].

The relationship between microcirculatory perfusion disturbances and clinical outcome remains unclear. Unfortunately, most studies included in this review did not provide data on clinical outcome. Moreover, remaining studies lacked detailed information concerning the extent of microcirculatory perfusion disturbances and outcome. Furthermore included studies were not powered sufficiently to draw any conclusions on the association between microcirculatory perfusion disturbances and important complications of cardiac surgery such as mediastinitis, stroke, acute kidney injury (AKI), acute lung injury, and mortality. However, ample literature exists on the correlation between microcirculatory perfusion disturbances and outcome in other settings such as sepsis and hemorrhagic shock [37, 38]. It was shown that reduced microvascular density at 72 h was independently associated with mortality in septic shock patients [43], whereas early improvement of microcirculatory perfusion parameters was seen in survivors [37]. Interestingly, also early goal-directed therapy may improve microcirculatory perfusion parameters irrespective of global hemodynamics and was found to be associated with reduced multi-organ failure [44]. It is likely similar relationships may be found in a cardiac surgery setting and future research should explore this correlation.

### Future perspectives

This review outlines the current evidence on the course of CPB-induced alterations in sublingual microcirculatory perfusion during cardiac surgery; however, the duration of this CPB-effect remains unclear. Further research is warranted to discover the full extent of CPB-induced microcirculatory perfusion disturbances and to identify strategies to improve the restoration capacity of the microvasculature. Also, the relationship between microcirculatory perfusion disturbances and clinical outcomes in cardiac surgery is unclear. A recent review by De Backer outlines the challenges that face implementation of hand-held microscopy into everyday clinical practice [45]. Microcirculatory imaging tools lack clearly defined endpoints and therapeutic interventions to reach these targets. Also, image analysis remains time consuming. Perhaps, the advent of machine learning in medicine will provide the necessary improvements to fully automate image analysis in the near future.

### Limitations

The effect of CPB on microcirculatory perfusion was not the primary objective of all included studies; therefore, these studies may not have been adequately powered for this analysis. Moreover, included studies were relatively small single-center studies. Heterogeneity of included studies makes this review susceptible to various forms of bias such as selection and confirmation bias. Despite guidelines to ensure uniform data reporting, this review contains 23 subtly different definitions of the accepted microvascular parameters measured at different time points and intervals with varying baselines. The CPB protocols, when reported, had considerable variation, which may potentially have influenced microcirculatory perfusion measurements.

Another limitation is that this review focusses solely on sublingual measurements of the microcirculatory perfusion. The sublingual area is easy to reach in the operating theater and afterwards on the ICU or ward, and the sublingual microcirculatory network shares the same embryologic origin as the gut. Both in the experimental [46, 47] and clinical [48, 49] setting, sublingual microcirculatory changes correlate well with microcirculatory changes in other tissues such as the gastric and gut mucosa, although this notion has been challenged in an ICU sepsis setting [50].

## Conclusion

Cardiopulmonary bypass during cardiac surgery reduces sublingual microcirculatory perfusion. These microcirculatory perfusion disturbances following cardiac surgery with cardiopulmonary bypass are mainly characterized by a decrease in the amount of perfused capillaries leading to reduced functional capillary density (FCD), perfused vessel density (PVD), or proportion of perfused vessels (PPV). In contrast, no effect of CPB on total vessel density (TVD), small vessel density (SVD), or vessel density (VD) was observed (Fig. 2). So far, data concerning the effect of CPB on microvascular flow index (MFI) remains conflicting. In conclusion, cardiopulmonary bypass during cardiac surgery mainly impairs microcirculatory flow, without affecting the overall amount of microvessels. This heterogeneity in microvascular flow is however not adequately reflected in MFI. Interestingly, four studies found CPB-induced microcirculatory perfusion disturbances lasted for at least 24 h after surgery, indicating a prolonged impairment of microcirculatory perfusion in the postoperative period. Further research is warranted to confirm these findings in larger groups and to identify the relationship with clinical outcome.

## Supplementary information

**Additional file 1: Supplemental methods.** Full search strategy.

**Additional file 2: Supplemental Table 1.** Study design and main findings of included studies.

**Additional file 3: Supplemental Table 2.** Patient characteristics of included studies.

**Additional file 4: Supplemental Table 3.** Quality assessment of included observational studies (3A) and randomized controlled trials (3B).

## Data Availability

All data generated or analyzed during this study are included in this published article [and its supplementary information files].

## Abbreviations

CPB: Cardiopulmonary bypass
ICU: Intensive care unit
OPS: Orthogonal polarization spectral imaging technique
SDF: Side-stream dark field imaging
IDF: Incidence dark field imaging
FCD: Functional capillary density
TVD: Total vessel density
PPV: Proportion of perfused vessels
PVD: Perfused vessel density
MFI: Microvascular flow index
VD: Vessel density
SVD: Small vessel density
CABG: Coronary artery bypass grafting
OPCABG: Off-pump coronary artery bypass grafting
ROS: Reactive oxygen species
NO: Nitric oxide
IFNy: Interferon gamma
TNFα: Tumor necrosis factor alpha
IL: Interleukin

## References

[1] D'Agostino RS, Jacobs JP, Badhwar V, Fernandez FG, Paone G, Wormuth DW (2018). The Society of Thoracic Surgeons Adult Cardiac Surgery Database: 2018 update on outcomes and quality. Ann Thorac Surg.

[2] Siregar S, de Heer F, Groenwold RH, Versteegh MI, Bekkers JA, Brinkman ES (2014). Trends and outcomes of valve surgery: 16-year results of Netherlands Cardiac Surgery National Database. Eur J Cardiothorac Surg.

[3] De Backer D, Creteur J, Dubois MJ, Sakr Y, Vincent JL (2004). Microvascular alterations in patients with acute severe heart failure and cardiogenic shock. Am Heart J.

[4] De Backer D, Creteur J, Preiser JC, Dubois MJ, Vincent JL (2002). Microvascular blood flow is altered in patients with sepsis. Am J Respir Crit Care Med.

[5] De Backer D, Ortiz JA, Salgado D (2010). Coupling microcirculation to systemic hemodynamics. Curr Opin Crit Care.

[6] Groner W, Winkelman JW, Harris AG, Ince C, Bouma GJ, Messmer K (1999). Orthogonal polarization spectral imaging: a new method for study of the microcirculation. Nat Med.

[7] Aykut G, Veenstra G, Scorcella C, Ince C, Boerma C (2015). Cytocam-IDF (incident dark field illumination) imaging for bedside monitoring of the microcirculation. Intensive Care Med Exp.

[8] Ince C, Boerma EC, Cecconi M, De Backer D, Shapiro NI, Duranteau J (2018). Second consensus on the assessment of sublingual microcirculation in critically ill patients: results from a task force of the European Society of Intensive Care Medicine. Intensive Care Med.

[9] Moher D, Liberati A, Tetzlaff J, Altman DG (2009). Preferred reporting items for systematic reviews and meta-analyses: the PRISMA statement. J Clin Epidemiol.

[10] National Heart L, and Blood Institute (NIH). . Study quality assessment tools [Available from: https://www.nhlbi.nih.gov/health-topics/study-quality-assessment-tools.

[11] De Backer D, Hollenberg S, Boerma C, Goedhart P, Buchele G, Ospina-Tascon G (2007). How to evaluate the microcirculation: report of a round table conference. Crit Care.

[12] Atasever B, Boer C, Goedhart P, Biervliet J, Seyffert J, Speekenbrink R (2011). Distinct alterations in sublingual microcirculatory blood flow and hemoglobin oxygenation in on-pump and off-pump coronary artery bypass graft surgery. J Cardiothorac Vasc Anesth.

[13] Bauer A, Kofler S, Thiel M, Eifert S, Christ F (2007). Monitoring of the sublingual microcirculation in cardiac surgery using orthogonal polarization spectral imaging: preliminary results. Anesthesiology..

[14] De Backer D, Dubois MJ, Schmartz D, Koch M, Ducart A, Barvais L (2009). Microcirculatory alterations in cardiac surgery: effects of cardiopulmonary bypass and anesthesia. Ann Thorac Surg.

[15] Dekker NAM, Veerhoek D, Koning NJ, van Leeuwen ALI, PWG E, van den Brom CE, et al. Postoperative microcirculatory perfusion and endothelial glycocalyx shedding following cardiac surgery with cardiopulmonary bypass. Anaesthesia. 2019;74(5):609–618. 10.1111/anae.14577 PMC6590376 30687934

[16] Donndorf P, Kühn F, Vollmar B, Rösner J, Liebold A, Gierer P (2012). Comparing microvascular alterations during minimal extracorporeal circulation and conventional cardiopulmonary bypass in coronary artery bypass graft surgery: a prospective, randomized study. J Thorac Cardiovasc Surg.

[17] Donndorf P, Park H, Vollmar B, Alms A, Steinhoff G, Kaminski A. Microvascular alterations during surgical aortic valve replacement utilizing minimal extracorporeal circulation and conventional cardiopulmonary bypass. Thorac Cardiovasc Surg. 2014;19(2):211–7. 10.1093/icvts/ivu131 24796334

[18] O’Neil MP, Fleming JC, Badhwar A, Guo LR (2012). Pulsatile versus nonpulsatile flow during cardiopulmonary bypass: microcirculatory and systemic effects. Ann Thorac Surg.

[19] O’Neil MP, Alie R, Guo LR, Myers ML, Murkin JM, Ellis CG (2018). Microvascular responsiveness to pulsatile and nonpulsatile flow during cardiopulmonary bypass. Ann Thorac Surg.

[20] Özarslan NG, Ayhan B, Kanbak M, Celebioglu B, Demircin M, Ince C, et al. Comparison of the effects of sevoflurane, isoflurane, and desflurane on microcirculation in coronary artery bypass graft surgery. J Cardiothorac Vasc Anesth 2012;26(5):791–798. 10.1053/j.jvca.2012.03.019 22592139

[21] Holmgaard F, Vedel AG, Ravn HB, Nilsson JC, Rasmussen LS. Impact of mean arterial pressure on sublingual microcirculation during cardiopulmonary bypass—Secondary outcome from a randomized clinical trial. Microcirculation (New York, NY : 1994). 2018;25(5). 10.1111/micc.12459 29754402

[22] Mohamed H, Hosny H, Tawadros Md P, Elayashy Md Desa Fcai M, El-Ashmawi Md H. Effect of dexmedetomidine infusion on sublingual microcirculation in patients undergoing on-pump coronary artery bypass graft surgery: a prospective randomized trial. J Cardiothorac Vasc Anesth 2018;33(2):334–340. 10.1053/j.jvca.2018.06.016 30075898

[23] Prestes I, Riva J, Bouchacourt JP, Kohn E, López A, Hurtado FJ (2016). Microcirculatory changes during cardiac surgery with cardiopulmonary bypass. Rev Esp Anestesiol Reanim.

[24] Koning NJ, Vonk AB, van Barneveld LJ, Beishuizen A, Atasever B, van den Brom CE (2012). Pulsatile flow during cardiopulmonary bypass preserves postoperative microcirculatory perfusion irrespective of systemic hemodynamics. J Appl Physiol (Bethesda, Md : 1985).

[25] Koning NJ, Vonk AB, Meesters MI, Oomens T, Verkaik M, Jansen EK (2013). Microcirculatory perfusion is preserved during off-pump but not on-pump cardiac surgery. J Cardiothorac Vasc Anesth.

[26] Koning NJ, Vonk AB, Vink H, Boer C (2015). Side-by-side alterations in glycocalyx thickness and perfused microvascular density during acute microcirculatory alterations in cardiac surgery. Microcirculation.

[27] Yuruk K, Bezemer R, Euser M, Milstein DM, de Geus HH, Scholten EW (2012). The effects of conventional extracorporeal circulation versus miniaturized extracorporeal circulation on microcirculation during cardiopulmonary bypass-assisted coronary artery bypass graft surgery. Interact Cardiovasc Thorac Surg.

[28] Bienz M, Drullinsky D, Stevens LM, Bracco D, Noiseux N (2016). Microcirculatory response during on-pump versus off-pump coronary artery bypass graft surgery. Perfusion.

[29] den Uil CA, Lagrand WK, Spronk PE, van Domburg RT, Hofland J, Lüthen C (2008). Impaired sublingual microvascular perfusion during surgery with cardiopulmonary bypass: a pilot study. J Thorac Cardiovasc Surg.

[30] Koning NJ, Simon LE, Asfar P, Baufreton C, Boer C (2014). Systemic microvascular shunting through hyperdynamic capillaries after acute physiological disturbances following cardiopulmonary bypass. Am J Physiol Heart Circ Physiol.

[31] Koning NJ, Atasever B, Vonk AB, Boer C (2013). Changes in microcirculatory perfusion and oxygenation during cardiac surgery with or without cardiopulmonary bypass. J Cardiothorac Vasc Anesth.

[32] Koning NJ, de Lange F, van Meurs M, Jongman RM, Ahmed Y, Schwarte LA (2018). Reduction of vascular leakage by imatinib is associated with preserved microcirculatory perfusion and reduced renal injury in a rat model of cardiopulmonary bypass. Br J Anaesth.

[33] Dekker NAM, van Meurs M, van Leeuwen ALI, Hofland HM, van Slyke P, Vonk ABA, et al. Vasculotide, an angiopoietin-1 mimetic, reduces pulmonary vascular leakage and preserves microcirculatory perfusion during cardiopulmonary bypass in rats. Br J Anaesth. 2018. 10.1016/j.bja.2018.05.049. 10.1016/j.bja.2018.05.049 30336848

[34] Dekker NAM, van Leeuwen ALI, van de Ven PM, de Vries R, Hordijk PL, Boer C, van den Brom CE (2020). Pharmacological interventions to reduce edema following cardiopulmonary bypass: a systematic review and meta-analysis. J Crit Care.

[35] Vellinga NA, Boerma EC, Koopmans M, Donati A, Dubin A, Shapiro NI (2015). International study on microcirculatory shock occurrence in acutely ill patients. Crit Care Med.

[36] Scorcella C, Damiani E, Domizi R, Pierantozzi S, Tondi S, Carsetti A (2018). MicroDAIMON study: Microcirculatory DAIly MONitoring in critically ill patients: a prospective observational study. Ann Intensive Care.

[37] Sakr Y, Dubois MJ, De Backer D, Creteur J, Vincent JL (2004). Persistent microcirculatory alterations are associated with organ failure and death in patients with septic shock. Crit Care Med.

[38] Tachon G, Harrois A, Tanaka S, Kato H, Huet O, Pottecher J (2014). Microcirculatory alterations in traumatic hemorrhagic shock. Crit Care Med.

[39] Koch M, De Backer D, Vincent JL, Barvais L, Hennart D, Schmartz D (2008). Effects of propofol on human microcirculation. Br J Anaesth.

[40] El Dib R, Guimaraes Pereira JE, Agarwal A, Gomaa H, Ayala AP, Botan AG (2017). Inhalation versus intravenous anaesthesia for adults undergoing on-pump or off-pump coronary artery bypass grafting: a systematic review and meta-analysis of randomized controlled trials. J Clin Anesth.

[41] Murphy GS, Hessel EA, Groom RC (2009). Optimal perfusion during cardiopulmonary bypass: an evidence-based approach. Anesth Analg.

[42] Hoefeijzers MP, Ter Horst LH, Koning N, Vonk AB, Boer C, Elbers PWG (2015). The pulsatile perfusion debate in cardiac surgery: answers from the microcirculation?. J Cardiothorac Vasc Anesth.

[43] Massey MJ, Hou PC, Filbin M, Wang H, Ngo L, Huang DT (2018). Microcirculatory perfusion disturbances in septic shock: results from the ProCESS trial. Crit Care.

[44] Trzeciak S, McCoy JV, Dellinger RP, Arnold RC, Rizzuto ML, Abate NL (2008). Early increases in microcirculatory perfusion during protocol-directed resuscitation are associated with reduced multi-organ failure at 24h in patients with sepsis. Intensive Care Med.

[45] De Backer D (2019). Is microcirculatory assessment ready for regular use in clinical practice?. Curr Opin Crit Care.

[46] Nakagawa Y, Weil MH, Tang W, Sun S, Yamaguchi H, Jin X (1998). Sublingual capnometry for diagnosis and quantitation of circulatory shock. Am J Respir Crit Care Med.

[47] Povoas HP, Weil MH, Tang W, Moran B, Kamohara T, Bisera J (2000). Comparisons between sublingual and gastric tonometry during hemorrhagic shock. Chest.

[48] Marik PE (2001). Sublingual capnography: a clinical validation study. Chest.

[49] Creteur J, De Backer D, Sakr Y, Koch M, Vincent JL (2006). Sublingual capnometry tracks microcirculatory changes in septic patients. Intensive Care Med.

[50] Boerma EC, van der Voort PH, Spronk PE, Ince C (2007). Relationship between sublingual and intestinal microcirculatory perfusion in patients with abdominal sepsis. Crit Care Med.

