# Supplementary material for: Microcirculatory perfusion disturbances following cardiopulmonary bypass: a systematic review
Source: Crit Care. 2020 May 13;24:218. doi: 10.1186/s13054-020-02948-w (PMC7222340; doi:10.1186/s13054-020-02948-w)
Supplement: Supplementary file 1 — Additional file 1: Supplemental methods. Full search strategy. [file 13054_2020_2948_MOESM1_ESM.docx]

**Microcirculatory perfusion disturbances following cardiopulmonary bypass: a systematic review**

**Additional file 1: Supplemental Methods - Search strategy**

**Pubmed; 170 hits**

(“Cardiopulmonary Bypass”[MeSH] OR cardiopulmonary bypass*[tiab] OR heart-lung bypass*[tiab] OR heart-lung machine*[tiab] OR cardio-pulmonary bypass*[tiab] OR CPB[tiab] OR heart-bypass*[tiab] OR “Cardiac Surgical Procedures”[MeSH] OR "Thoracic Surgery"[Mesh] OR cardiac surgical procedure*[tiab] OR cardiac surger*[tiab] OR heart surgical procedure*[tiab] OR heart surger*[tiab] OR CABG[tiab] OR "coronary artery bypass grafting"[tiab]) AND (“Microcirculation”[MeSH] OR microcirculation[tiab] OR “microcirculatory”[tiab] OR “microcirculatory perfusion”[tiab] OR “microperfusion”[tiab] OR “Blood flow velocity”[MeSH] OR “Blood flow velocity”[tiab] OR “orthogonal polarization spectral*”[tiab] OR OPS[tiab] OR “side stream dark field”[tiab] OR SDF[tiab] OR “incidence dark field”[tiab] OR IDF[tiab] OR “proportion of perfused vessels”[tiab] OR PPV[tiab OR “perfused vessel density”[tiab] OR PVD[tiab] OR “total vessel density”[tiab] OR TVD[tiab])

**Embase; 107 hits**

('microcirculation'/exp OR microcirculation*:ti,ab,kw OR 'blood flow velocity'/exp OR 'blood flow velocity':ti,ab,kw OR 'microvasculature'/exp OR microvasculature:ti,ab,kw OR microcirculatory:ti,ab,kw OR 'microcirculatory perfusion':ti,ab,kw OR microperfusion:ti,ab,kw) AND (ops:ti,ab,kw OR 'orthogonal polarization spectral':ti,ab,kw OR sdf:ti,ab,kw OR 'side stream dark field':ti,ab,kw OR idf:ti,ab,kw OR 'incidence dark field':ti,ab,kw OR ppv:ti,ab,kw OR 'proportion of perfused vessels':ti,ab,kw OR pvd:ti,ab,kw OR 'perfused vessel densit*':ti,ab,kw OR 'total vessel densit*':ti,ab,kw OR tvd:ti,ab,kw) AND ('heart surgery'/exp OR 'cardiac surgical procedure*':ti,ab,kw OR 'cardiac surger*':ti,ab,kw OR 'heart surgical procedure*':ti,ab,kw OR 'heart surger*':ti,ab,kw OR cabg:ti,ab,kw OR 'coronary artery bypass grafting':ti,ab,kw OR 'cardiopulmonary bypass'/exp OR 'cardiopulmonary bypass*':ti,ab,kw OR 'heart-lung bypass'/exp OR 'heart lung bypass*':ti,ab,kw OR 'heart lung machine'/exp OR 'heart lung machine*':ti,ab,kw OR cpb:ti,ab,kw OR 'heart-bypass':ti,ab,kw)
