# Supplementary material for: Microcirculatory perfusion disturbances following cardiopulmonary bypass: a systematic review
Source: Crit Care. 2020 May 13;24:218. doi: 10.1186/s13054-020-02948-w (PMC7222340; doi:10.1186/s13054-020-02948-w)
Supplement: Supplementary file 2 — Additional file 2: Supplemental Table 1. Study design and main findings of included studies. [file 13054_2020_2948_MOESM2_ESM.docx]

| **Supplemental table 1.** Main findings per study | | | | |  |
| --- | --- | --- | --- | --- | --- |
| **Study** | **Type of study** | **Imaging technique** | **Endpoints** | **Main finding** | |
| Atasever 2011 | Observational study | SDF | FCD, EV, uHbO2 | On-pump and off-pump cardiac surgery are associated with distinct alterations in sublingual microcirculatory perfusion and hemobglobin oxygenation. | |
| Bauer 2007 | Observational study | OPS | FCD, EV, MD | Orthogonal polarization spectral imaging revealed no major changes of microvascular perfusion during uncomplicated hypothermic CPB. | |
| Bienz 2016 | Observational study | SDF | TVD, PVD, SVD | The results do not show a marked preservation of the microcirculation during and after OPCABG compared to ONCABG. As fluctuations in the microvascular parameters coincide with temperature fluctuations this might be a confounding factor. | |
| De Backer 2009 | Observational study | OPS | VD, PVD, PPV | Microcirculatory alterations are observed in cardiac surgery patients whether or not CPB is used. Anesthesia contributes to these alterations, but its effects are transient. | |
| Dekker 2019 | Observational study | SDF | PVD, PPV, PBR, plasma heparan sulphate, plasma syndecan-1 | Cardiopulmonary bypass-induced acute microcirculatory perfusion disturbances persist in the first three postoperative days, and are associated with prolonged endothelial glycocalyx shedding. | |
| Den Uil 2008 | Observational study | SDF | MFI | Median microvascular flow index of medium vessels decreased after starting CPB. Decreases in microvascular flow index occurred irrespective of changes in systemic blood pressure. After each patient's return to the ICU, microvascular flow index increased and normalized in all microvessels. | |
| Donndorf 2012 | Randomized controlled trial | OPS | FCD, EV, MD | Orthogonal polarization spectral imaging data reveal an impairment of microvascular perfusion during on-pump CABG. Changes in FCD indicate a faster recovery of the microvascular perfusion in MECC during the reperfusion period. | |
| Donndorf 2014 | Randomized controlled trial | OPS | FCD, EV, MD | The use of MECC in AVR did not affect procedural safety and resulted in beneficial preservation of microvascular blood flow velocity and significantly reduced haemodilution during CPB. In contrast to CABG surgery, the use of MECC did not improve FCD during surgical AVR. | |
| Holmgaard 2018 | Randomized controlled trial | SDF | TVD, MFI, PVD, PPV, HI | We found no significant difference in sublingual microcirculatory flow expressed as MFI according to 2 different levels of MAP during CPB. | |
| Koning 2012 | Randomized controlled trial | SDF | TVD, MFI, PVD | Pulsatile cardiopulmonary bypass preserves microcirculatory perfusion throughout the early postoperative period, irrespective of systemic hemodynamics. | |
| Koning 2013 | Observational study | SDF | TVD, MFI, PVD | Microcirculatory perfusion remained unaltered throughout off-pump surgery. In contract, microvascular perfusion declined after initiation of cardiopulmonary bypass and did not recover in the early postoperative phase. | |
| Koning 2014 | Observational study | SDF | MFI, EV, HI | The current study provides the first direct human evidence for a microvascular shunting phenomenon through hyperdynamic capillaries following acute physiological disturbances after onset of CPB. | |
| Koning 2015 | Observational study | SDF | PVD, PBR | Endothelial glycocalyx dimensions decrease after onset of CPB and are closely related to microvascular perfusion when assessed with a novel, noninvasive technique. | |
| Mohamed 2018 | Randomized controlled trial | SDF | TVD, MFI, PVD, PPV | This trial demonstrated that dexmedetomidine infusion improved sublingual microcirculation indices in patients undergoing on-pump CABG surgery. | |
| O'Neil 2012 | Randomized controlled trial | OPS | PPV | Pulsatile perfusion is superior to nonpulsatile perfusion at preserving the microcirculation. | |
| O'Neil 2018 | Randomized controlled trial | OPS | PPV | Pulsatility generated by the roller pump during CPB improves microcirculatory blood flow and tissue oxygen saturation compared with nonpulsatile flow in high-risk cardiac surgical patients. | |
| Özarslan 2012 | Randomized controlled trial | OPS | TVD, MFI, PVD, PPV | Sevoflurane had a negative effect on the microcirculation. Isoflurane decreased vascular density and increased flow. Desflurane produced stable effects on the microcirculation. | |
| Prestes 2016 | Observational study | SDF | VD, MFI, PVD, PPV,HI | In patients with intermdiate/high preoperative risk, cardiac surgery and CPB can involve an increase in MFI and blood lactate at the end of the study. | |
| Yuruk 2012 | Randomized controlled trial | SDF | MFI, PVD, neutrophil gelatinase-associated lipocalin, creatinine, creatinine clearance | The use of the miniaturized extracorporeal circulation system is associated with a statistically significant (but clinically insignificant) reduction in haemodilution and microcirculatory hypoperfusion compared with the use of the conventional extracorporeal circulation system. | |

CPB; cardiopulmonary bypass, SDF; side-stream dark field imaging, OPS; orthogonal polarization spectral imaging, N, number of participants; CABG; coronary artery bypass grafting, FCD; functional capillary density, VD; vessel density, TVD; total vessel density, PVD; perfused vessel density, PPV, proportion of perfused vessels, MFI; microvascular flow index, PBR; perfused boundary region, HI; heterogeneity index.
