# Supplementary material for: Microcirculatory perfusion disturbances following cardiopulmonary bypass: a systematic review
Source: Crit Care. 2020 May 13;24:218. doi: 10.1186/s13054-020-02948-w (PMC7222340; doi:10.1186/s13054-020-02948-w)
Supplement: Supplementary file 3 — Additional file 3: Supplemental Table 2. Patient characteristics of included studies. [file 13054_2020_2948_MOESM3_ESM.docx]

| **Supplemental table 2.** Patient characteristics per study | | | | | | | | | |
| --- | --- | --- | --- | --- | --- | --- | --- | --- | --- |
| **Study** | **N** | **Groups** | **N** | **Age** | **Male (%)** | **BSA** | **BMI** | **Hypertension (%)** | **Diabetes (%)** |
| Atasever 2011 | 48 | 1. CABG SDF imaging | 12 | 71 ± 7 | 6 (50%) | 1.9 ± 0.1 | NR | 2 (17%) | 3 (25%) |
|  |  | 2. CABG RS imaging | 12 | 70 ± 8 | 5 (42%) | 1.8 ± 0.2 | NR | 3 (25%) | 3 (25%) |
|  |  | 3. OPCABG SDF imaging | 12 | 65 ± 9 | 9 (75%) | 2.0 ± 0.1 | NR | 3 (25%) | 3 (25%) |
|  |  | 4. OPCABG RS imaging | 12 | 67 ± 12 | 6 (50%) | 1.9 ± 0.2 | NR | 5 (42%) | 4 (33%) |
| Bauer 2007 | 47 | 1. Cardiac surgery with CPB | 47 | 66 (59-74) | 32 (68%) |  | 26.6 (24.5-29.7) | 20 (43%) | 15 (32%) |
| Bienz 2016 | 32 | 1. CABG | 16 | 68.5 ± 7.8 | 13 (81%) | 1.8 ± 0.5 | 30.0 ± 5.1 | 14 (88%) | 5 (31%) |
|  |  | 2. OPCABG | 16 | 68.5 ± 10.0 | 11 (69%) | 1.9 ± 0.2 | 28.7 ± 5.5 | 13 (81%) | 9 (56%) |
| De Backer 2009 | 17 | 1. Cardiac surgery with CPB | 9 | 66 (55-75) | 6 (67%) | NR | NR | 5 (56%) | 4 (44%) |
|  |  | 2. OPCABG | 6 | 66 (65-70) | 6 (100%) | NR | NR | 3 (50%) | 3 (50%) |
| Dekker 2019 | 17 | 1. CABG | 17 | 69 (63-74) | 15 (88%) | NR | 29 | 5 (29%) | 2 (12%) |
| Den Uil 2008 | 25 | 1. Cardiac surgery with CPB | 25 | 65 (61-74) | 20 (80%) | NR | NR | 17 (68%) | 6 (24%) |
| Donndorf 2012 | 40 | 1. CABG | 20 | 69 ± 8 | 16 (80%) | NR | NR | NR | 11 (55%) |
|  |  | 2. CABG with MECC | 20 | 67 ± 8 | 14 (70%) | NR | NR | NR | 9 (45%) |
| Donndorf 2014 | 20 | 1. AVR | 10 | 65 ± 12 | 4 (40%) | NR | NR | NR | 1 (10%) |
|  |  | 2. AVR with MECC | 10 | 69 ± 11 | 5 (50%) | NR | NR | NR | 3 (30%) |
| Holmgaard 2018 | 30 | 1. CABG with HMAP | 16 | 68 ± 8 | 14 (88%) | NR | 28.0 ± 4.7 | 14 (88%) | 4 (25%) |
|  |  | 2. CABG with LMAP | 14 | 65 ± 7 | 14 (100%) | NR | 27.9 ± 3.4 | 12 (86%) | 2 (14%) |
| Koning 2012 | 33 | 1. CABG non pulsatile CPB | 17 | 65 ± 7 | 14 (82%) | 2.1 ± 0.2 | NR | 14 (82%) | 2 (12%) |
|  |  | 2. CABG pulsatile CPB | 16 | 67 ± 11 | 10 (63%) | 1.9 ± 0.2 | NR | 14 (88%) | 4 (25%) |
| Koning 2013 | 26 | 1. CABG | 13 | 65 (62-72) | 12 (92%) | 2.0 ± 0.2 | NR | NR | 2 (15%) |
|  |  | 2. OPCABG | 13 | 63 (54-73) | 13 (100%) | 2.0 ± 0.2 | NR | NR | 1 (8%) |
| Koning 2014 | 31 | 1. CABG | 18 | 68 ± 8 | 15 (83%) | 2.0 ± 0.2 | NR | NR | NR |
|  |  | 2. OPCABG | 13 | 62 ± 9 | 12 (92%) | 2.0 ± 0.2 | NR | NR | NR |
| Koning 2015 | 36 | 1. OPCABG | 12 | 65 (54-65) | NA | NR | NR | NR | 1 (8%) |
|  |  | 2. CABG non pulsatile CPB | 12 | 65 (61-69) | NA | NR | NR | NR | 2(16%) |
|  |  | 3. CABG pulsatile CPB | 12 | 74 (71-75) | NA | NR | NR | NR | 2(16%) |
| Mohamed 2018 | 70 | 1. CABG with regular anesthesia | 35 | 59 ± 4 | 29 (83%) | NR | NR | 15 (43%) | 10 (29%) |
|  |  | 2. CABG with dexmedetomidine | 35 | 58 ± 7 | 23 (66%) | NR | NR | 19 (54%) | 19 (54%) |
| O'Neil 2012 | 20 | 1. Cardiac surgery pulsatile CPB | 10 | 75.3 ± 7.0 | 5 (50%) | 1.90 ± 0.33 | NR | 9 (90%) | 4 (40%) |
|  |  | 2. Cardiac surgery non pulsatile CPB | 10 | 69.7 ± 11.8 | 7 (70%) | 1.84 ± 0.30 | NR | 7 (70%) | 2 (20%) |
| O'Neil 2018 | 20 | 1. Cardiac surgery pulsatile CPB | 10 | 72.4 ± 14.5 | 5 (50%) | 2.03 ± 0.19 | NR | 7 (70%) | 2 (20%) |
|  |  | 2. Cardiac surgery non pulsatile CPB | 10 | 74.6 ± 9.9 | 7 (70%) | 1.91 ± 0.26 | NR | 8 (80%) | 2 (20%) |
| Özarslan 2012 | 30 | 1. CABG with sevoflurane | 10 | 67.7 ± 4.6 | 9 (90%) | NR | NR | NR | NR |
|  |  | 2. CABG with isoflurane | 10 | 59.6 ± 11.2 | 8 (80%) | NR | NR | NR | NR |
|  |  | 3. CABG with desflurane | 10 | 61.0 ± 10.1 | 8 (80%) | NR | NR | NR | NR |
| Prestes 2016 | 22 | 1. Cardiac surgery with CPB | 22 | 64.3 ± 15.3 | 7 (32%) | NR | NR | NR | NR |
| Yuruk 2012 | 20 | 1. CABG | 10 | 64 ± 8 | 9 (90%) | NR | 28 ± 4 | 3 (30%) | 2 (20%) |
|  |  | 2. CABG with MECC | 10 | 66 ± 9 | 9 (90%) | NR | 27 ± 3 | 2 (20%) | 1 (10%) |

CPB; cardiopulmonary bypass, SDF; side-stream dark field imaging, OPS; orthogonal polarization spectral imaging, N, number of participants; CABG; coronary artery bypass grafting, MECC; minimal extracorporeal circulation.
