# Supplementary material for: Microcirculatory perfusion disturbances following cardiopulmonary bypass: a systematic review
Source: Crit Care. 2020 May 13;24:218. doi: 10.1186/s13054-020-02948-w (PMC7222340; doi:10.1186/s13054-020-02948-w)
Supplement: Supplementary file 4 — Additional file 4: Supplemental Table 3. Quality assessment of included observational studies (3A) and randomized controlled trials (3B). [file 13054_2020_2948_MOESM4_ESM.docx]

| **Supplemental table 3A.** Quality assessment of included observational studies | | | | | | | | | | | |  |  |  |  |
| --- | --- | --- | --- | --- | --- | --- | --- | --- | --- | --- | --- | --- | --- | --- | --- |
| **Study** | **1** | **2** | **3** | **4** | **5** | **6** | **7** | **8** | **9** | **10** | **11** | | **12** | **13** | **14** |
| Atasever 2011 | Yes | Yes | NA | Yes | No | Yes | Yes | NA | No | NA | Yes | | Not clear | Yes | No |
| Bauer 2007 | Yes | Yes | NA | Yes | No | Yes | Yes | NA | Yes | NA | Yes | | Not clear | Yes | No |
| Bienz 2016 | Yes | Yes | NA | Yes | No | Yes | Yes | NA | Yes | NA | Yes | | Not clear | Yes | No |
| De Backer 2009 | Yes | Yes | NA | Yes | Yes | Yes | Yes | NA | Yes | NA | Yes | | Yes | Yes | No |
| Dekker 2019 | Yes | Yes | NA | Yes | Yes | Yes | Yes | NA | Yes | NA | Yes | | Yes | Yes | No |
| Den Uil 2008 | Yes | Yes | NA | No | Yes | Yes | Yes | NA | Yes | NA | Yes | | Not clear | Yes | No |
| Koning 2013 | Yes | Yes | NA | Yes | Yes | Yes | Yes | NA | Yes | NA | Yes | | Yes | Yes | No |
| Koning 2014 | Yes | Yes | NA | Yes | Yes | Yes | Yes | NA | Yes | NA | Yes | | Yes | Yes | No |
| Koning 2015 | Yes | Yes | NA | Yes | Yes | Yes | Yes | NA | Yes | NA | Yes | | Yes | Yes | No |
| Prestes 2016 | Yes | Yes | NA | Yes | Yes | Yes | Yes | NA | No | NA | Yes | | Yes | Yes | No |

1. Was the research question or objective in this paper clearly stated?

2. Was the study population clearly specified and defined?

3. Was the participation rate of eligible persons at least 50%?

4. Were all the subjects selected or recruited from the same or similar populations (including the same time period)? Were inclusion and exclusion criteria for being in the study prespecified and applied uniformly to all participants?

5. Was a sample size justification, power description, or variance and effect estimates provided?

6. For the analyses in this paper, were the exposure(s) of interest measured prior to the outcome(s) being measured?

7. Was the timeframe sufficient so that one could reasonably expect to see an association between exposure and outcome if it existed?

8. For exposures that can vary in amount or level, did the study examine different levels of the exposure as related to the outcome (e.g., categories of exposure, or exposure measured as continuous variable)?

10. Was the exposure(s) assessed more than once over time?

11. Were the outcome measures (dependent variables) clearly defined, valid, reliable, and implemented consistently across all study participants?

12. Were the outcome assessors blinded to the exposure status of participants?

14. Were key potential confounding variables measured and adjusted statistically for their impact on the relationship between exposure(s) and outcome(s)?

| **Supplemental table 3B.** Quality assessment of included randomized controlled trials | | | | | | | | | | | | | | |
| --- | --- | --- | --- | --- | --- | --- | --- | --- | --- | --- | --- | --- | --- | --- |
| **Study** | **1** | **2** | **3** | **4** | **5** | **6** | **7** | **8** | **9** | **10** | **11** | **12** | **13** | **14** |
| Donndorf 2012 | Yes | Yes | Yes | NA | Yes | Yes | Yes | Yes | Yes | Yes | Yes | Yes | Yes | Yes |
| Donndorf 2014 | Yes | Yes | Yes | NA | Yes | Yes | Yes | Yes | Yes | Yes | Yes | No | Yes | Yes |
| Holmgaard 2018 | Yes | Yes | Yes | NA | Yes | Yes | Yes | Yes | Yes | Yes | Yes | No | Yes | No |
| Koning 2012 | Yes | Yes | Yes | NA | Unclear | Yes | Yes | Yes | Yes | Yes | Yes | No | Yes | Yes |
| Mohamed 2018 | Yes | Unclear | Unclear | NA | Yes | Yes | Yes | Yes | Yes | Yes | No | Yes | Yes | Yes |
| O'Neil 2012 | Yes | Yes | Yes | NA | Yes | Yes | Yes | Yes | Yes | Yes | Yes | No | Yes | Yes |
| O'Neil 2018 | Yes | Yes | Yes | NA | Yes | Yes | Yes | Yes | Yes | Yes | Yes | No | Yes | Yes |
| Özarslan 2012 | Yes | Yes | Yes | NA | Yes | Yes | Yes | Yes | Yes | Yes | Yes | Yes | Yes | Yes |
| Yuruk 2012 | Yes | Unclear | No | NA | Unclear | Yes | Yes | Yes | Yes | No | Yes | No | Yes | Yes |

1. Was the study described as randomized, a randomized trial, a randomized clinical trial, or and RCT?

2. Was the method of randomization adequate (i.e., use of randomly generated assignment)?

4. Were study participants and providers blinded to treatment group assignment?

5. Were the people assessing the outcomes blinded to the participants' group assignments?

6. Were the groups similar at baseline on important characteristics that could affect outcomes (e.g., demographics, risk factors, co-morbid conditions)?

7. Was the overall drop-out rate from the study at endpoint 20% or lower of the number allocated to treatment?

8. Was the differential drop-out rate (between treatment groups) at endpoint 15 percentage points or lower?

9. Was there high adherence to the intervention protocols in each treatment group?

10. Were other interventions avoided or similar in the groups (e.g., similar background treatments)?

11. Were outcomes assessed using valid and reliable measures, implemented consistently across all study participants?

12. Did the authors report that the sample size was sufficiently large to be able to detect a difference in the main outcome between groups with at least 80% power?

13. Were outcomes reported or subgroups analyzed prespecified (i.e. identified before analyses were conducted)?

14. Were all randomized participants analyzed in the group to which they were originally assigned, i.e., did the use an intention to treat analysis?
